# Supplementary material for: Sex-differences in prostaglandin signaling: a semi-systematic review and characterization of PTGDS expression in human sensory neurons
Source: Sci Rep. 2023 Mar 22;13:4670. doi: 10.1038/s41598-023-31603-x (PMC10033690; doi:10.1038/s41598-023-31603-x)
Supplement: Supplementary file 5 — Supplementary Legends. [file 41598_2023_31603_MOESM5_ESM.docx]

**Supplementary files:**

**Suppl. File 1**: **Review and summary of preclinical studies included in semi-systematic review.** **Tab A** – Studies included in semi-systematic review and the biological sex of the animals/subjects (in chronological order). **Tab B** – Details for studies that analyzed data separated by sex. Columns: (1) Paper: Year, first author and PMID of article. (2) Prostaglandins: Specific prostaglandins measured, analyzed, or otherwise mentioned in the paper. (3) Cyclooxygenases: Specific cyclooxygenases measured, analyzed, or otherwise mentioned in the paper. (4) Receptors: Specific prostaglandin receptors studied or otherwise mentioned in the paper. (5) Species: Species of the animal model, including tissues, or human participants, including human tissues, used. (6) Sex: Sex of the study subjects or animals. (7) Number/description: Number of subjects included in the study and characteristics of study subjects or animal models (8) Model: Experimentally induced pain model or clinically occurring pain disease model used. (9) Tissue: Tissues sampled, evaluated, or used in analyses in the study. (10) Injection/Drug administration route: Site of injections or route of administration of drugs. (11) Identified Sex Differences: “No” if analysis separated by sex revealed no mechanistic or functional sex-differences; “Yes” if analysis separated by sex revealed mechanistic and/or functional sex differences.

**Suppl. File 2**: **Number of preclinical studies including male, female or both sexes.**

**Suppl. File 3**: **Review and summary of clinical studies included in semi-systematic review.** Details for all studies included in semi-systematic review (not in chronological order). Columns: (1) Paper: Year, first author and PMID of article. (2) Prostaglandins: Specific prostaglandins measured, analyzed, or otherwise mentioned in the paper. (3) Cyclooxygenases: Specific cyclooxygenases measured, analyzed, or otherwise mentioned in the paper. (4) NSAIDs: Specific NSAIDs measured, analyzed, or otherwise mentioned in the paper. (5) Receptors: Specific prostaglandin receptors studied or otherwise mentioned in the paper. (6) Species: Species of the animal model, including tissues, or human participants, including human tissues, used. (7) Sex: Sex of the study subjects or animals. (8) Number/description: Number of subjects included in the study and characteristics of study subjects or animal models (9) Model: Experimentally induced pain model or clinically occurring pain disease model used. (10) Tissue: Tissues sampled, evaluated, or used in analyses in the study. (11) Injection/Drug administration route: Site of injections or route of administration of drugs. (12) Identified Sex Differences: “N/A” (Not Applicable) if only males, only females, or both males and females without sex as a biological variable were included; “No” if analysis separated by sex revealed no mechanistic or functional sex-differences; “Yes” if analysis separated by sex revealed mechanistic and/or functional sex differences.

**Suppl. file 4**: **Number of clinical studies including male subjects, female subjects or both sexes.**
